# Intraindividual Left–Right Side Differences of Sagittal Condylar Inclination (SCI) in Different Skeletal Classes

**DOI:** 10.3390/healthcare11091341

**Published:** 2023-05-07

**Authors:** Andi Ciprian Dragus, Augustin Mihai, Gabriela Tanase, Mihai Burlibasa, Corina Marilena Cristache

**Affiliations:** 1 Oral Implantology and Gnathology Department, Dr. Dragus Clinic, 110a Stirbei Voda Street, 010109 Bucharest, Romania; office@clinicadrdragus.ro; 2 Doctoral School, “Carol Davila” University of Medicine and Pharmacy, 37 Dionisie Lupu Street, 020021 Bucharest, Romania; augustin.mihai@umfcd.ro; 3 Implant Prosthetic Therapy, “Carol Davila” University of Medicine and Pharmacy, 19 Plevnei Ave., 010221 Bucharest, Romania; gabriela.tanase@umfcd.ro; 4 Department of Dental Techniques, “Carol Davila” University of Medicine and Pharmacy, 8, Eroilor Sanitari Blvd, 050474 Bucharest, Romania; mihai.burlibasa@umfcd.ro

**Keywords:** sagittal condylar inclination, condylography, temporomandibular joint, skeletal class, cone beam computed tomography

## Abstract

(1) Background: The temporomandibular joint (TMJ) is the most complex and one of the most important joints in the human body due to its essential roles in mastication, swallowing, breathing and speech. Several instruments have been used to track mandibular movements and register the characteristic parameters of the TMJ, among which condylography instruments are validated for the accurate clinical registration of the condylar path. Sagittal condylar inclination (SCI) is one of the most important parameters, together with the Bennett angle and the immediate side shift, used for articular settings in the process of oral rehabilitation. The aim of the present study was to evaluate the differences between the left and right SCI and to assess whether the differences were statistically significant for skeletal class, age, gender, dentate status, TMJ pathology or parafunctional habits. (2) Methods: One hundred and forty consecutive patients, fully dentate or partially edentulous, and with angle class I, II and II, were recruited. Their left and right SCIs were determined with an ultrasonic jaw tracking device. Each subject had to make three protrusive movements and three right and left laterotrusive movements. The software calculated the SCI from the mean of the protrusive movements. (3) Results: The mean values obtained for the right and left SCI were 34.68° (±12.44°) and 34.94° (±13.23°), respectively, with no statistically significant differences between the left and right values for gender, dentate status, TMJ disorders or parafunctional habits. Skeletal class III subjects registered lower SCI means, which were statistically significant for the left SCI. (4) Conclusions: For an optimal functionalization of prosthetic restorations and for an ideal treatment plan, the registration of both the left and right paths of the condyles and the articular disc should be taken into consideration.

## 1. Introduction

The temporomandibular joint (TMJ) is the most complex and one of the most important joints in the human body due to its essential roles in mastication, swallowing, breathing and speech [1,2]. The temporomandibular apparatus, consisting of the two TMJs and the associated neuromuscular system, guides mandibular movement, which occurs as a complex series of interrelated 3D rotational and translational activities [3].

The mandible is the only bone articulated to the cranial base with two, ideally symmetrical, joints, moving simultaneously, each composed of the glenoid fossa of the temporal bone, the condylar head of the mandible and the articular cartilage and disc. The TMJ is divided by the articular disc in two compartments lower, between the condyle and the inferior surface of the disc, where mostly rotation movements occur, and upper, between the superior surface of the disc and the fossa, where mostly translation movements take place [1,2,4].

TMJ pathologies, similarly to conditions affecting other joints in the body, are classified as: developmental anomalies, arthropathies, inflammatory arthritis, infective arthritis, neoplasia, metabolic disease, synovial disease, miscellaneous conditions (Paget’s disease of bone, acromegaly) and traumatic issues [5,6,7,8].

Maintaining adequate TMJ function is essential for a good quality of life. A loss of mandibular mobility, frequently associated with pain, intermittent joint sounds and masticatory muscle tenderness, characterizes temporomandibular disorders (TMD), which have a negative influence on an individual’s physical and mental health, affecting their work, social activities, diet and leading to affective and cognitive imbalances [9,10,11].

Investigating TMJ motion is very important for digital or analog articulator adjustment [12], for the diagnosis and screening of TMD, the fabrication of dentures and prosthodontic restorations, before orthodontic or orthognathic treatment [13], for assessing therapeutic measures, such as occlusal splints or TMJ arthroplasty, and so on [10].

The anatomy and dynamics of the TMJ are determinant factors in teeth morphology [14] and the functionality of the dento-maxillar apparatus. The characteristics of both TMJs and the pattern of mandibular movements needs to be determined for the maintenance and restoration of essential functions, such as mastication, speech, deglutition and esthetics.

Several instruments are used to track mandibular movements and register the characteristic parameters of TMJ. These instruments were classified by Woodford et al. into four categories: mechanical linkage systems, magnetic tracking systems, video motion analysis and radiographic tracking [10]. Mechanical linkage systems are the first and most utilized instruments used to register mandibular movements, and are mainly composed of a facebow and a registration device. Magnetic tracking systems and video motion analysis use sensors mounted on specific landmarks located on teeth structures or the face for acquiring kinematic data on the mandibular movements. Radiographic tracking, including video X-ray fluoroscopy and 4D computed tomography, records mandibular movements by registering the trajectory of radiopaque markers during a CBCT scan. Due to the high X-ray dose and the long-term radiation exposure required for dynamic registrations, these methods are approved only for research purposes [15].

Among the mandibular tracking tools, mechanical linkage systems are evidence-based instruments. They initially had bi-dimensional registration capacity, but now, due to digitalization, the tridimensional registration of complex mandibular movements is possible [10].

The main type of instruments included in this group are condylography instruments, previously known as axiography, which are devices for clinical functional analysis and the recording of the condylar pathway [2].

Condylography instruments are validated for the accurate registration of the path of the condyles and the articular disc traversing along the slope of the articular eminence (condylar guidance) [12,16]. These type of movements, depending on the shape of the mandibular fossa, the disc, the associated ligaments, the neuromuscular system, teeth morphology and the articular eminence, are registered by the measuring instrument as the sagittal condylar inclination (SCI) [17]. Besides the Bennett angle and the immediate side shift (ISS), SCI is one of the most important parameters used for articulator settings in the process of oral rehabilitation.

Most mechanical and virtual articulators use a single SCI value for the left and right TMJ, without considering the occurrence of intraindividual variations. The aim of the present study was to evaluate differences between the left and right SCI and to assess whether the differences were statistically significant for skeletal class, age, gender, dentate status, TMJ pathology or parafunctional habits.

## 2. Materials and Methods

This study was conducted in compliance with the World Medical Association’s Declaration of Helsinki, the Belmont report, the Council for International Organizations of Medical Sciences’ (CIOMS) guidelines and the International Conference on Harmonization in Good Clinical Practice (ICH-GCP). One hundred and forty consecutive patients were recruited from the “Carol Davila” University of Medicine and Pharmacy, Bucharest, and the Oral Implantology and Gnathology Department, Dr. Dragus Clinic, Bucharest. This study’s protocol was approved by the bioethics committee of the “Carol Davila” University of Medicine and Pharmacy (No. 11385/07.05.2021).

The inclusion criteria were: (1) age > 18 years old, (2) agreeing to participate in this study and sign the informed consent form, (3) no previous orthodontic treatments or cranio-facial surgery, (4) patients with complete dentition or who were partially edentulous with a stable maximum intercuspal position (MIC), (5) the persistence of natural/restored anterior teeth at least to the first mandibular premolar and (6) the requirement of a cone beam computed tomography (CBCT) investigation for orthodontic treatment or dental implants’ insertion.

The exclusion criteria were: (1) a limited mouth opening, (2) a history of Parkinson’s disease (which makes it impossible to perform an accurate CBCT or condylography), (3) severe systemic diseases, (4) undergoing pharmacological therapy with drugs that might have affected their psycho-physical condition or (5) acute TMJ pain.

All the patients gave their written informed consent for this study’s protocol.

A preliminary clinical examination was performed by a single well-trained operator (A.D.) and data regarding the degree of mandibular motion, mouth opening, parafunctional habits (such as bruxism), unilateral chewing habits, masticatory muscle tenderness during palpation, any TMJ pain or disfunction were collected. During an intraoral examination, patients were classified as fully dentate or partially edentulous.

### 2.1. Skeletal Class

**Figure 1:**
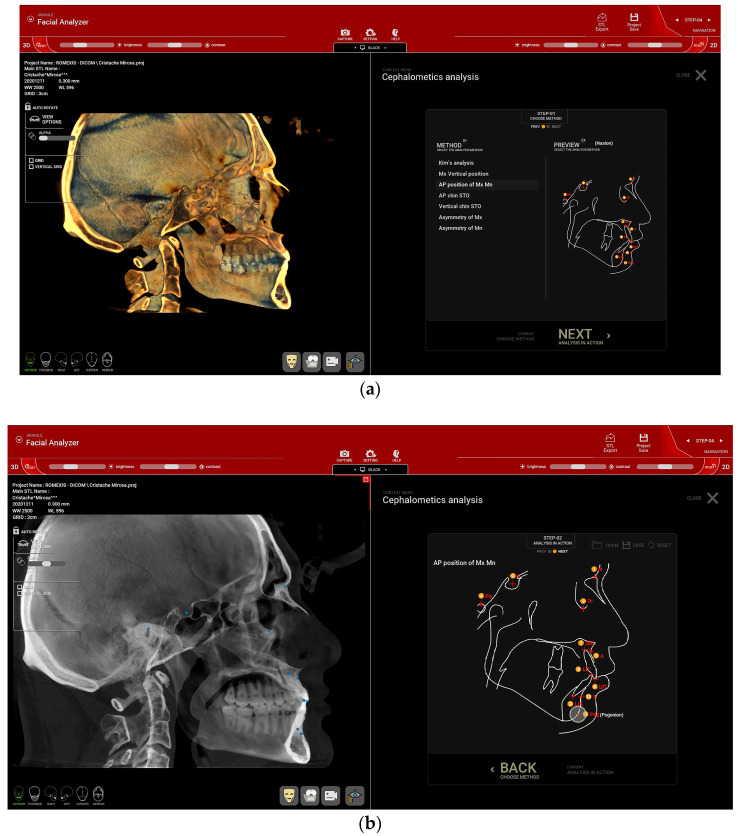
Cephalometric analysis of the CBCT in the R2Gate™ v 2.0 software. (**a**) The “AP position of Mx Mn” (antero-posterior position of the maxilla and mandible) option was selected. (**b**) Red dots within the image on the right (corresponding to the blue dots on the sagittal view of the patient’s CBCT) highlight the landmarks used, in the following order: nasion (N), sella (S), orbitale (Or), subspinale (A), upper incisor root apex (UIA), upper incisor incisal edge (UIT), lower incisor incisal edge (LIT), lower incisor root apex (LIA), supramentale (B) and pogonion (Pog).

**Figure 2:**
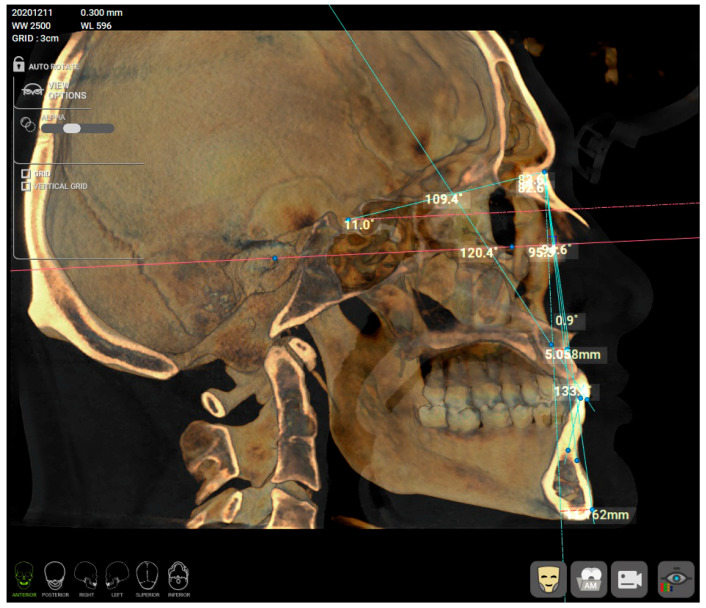
After marking the corresponding anthropometric reference points, the software automatically calculated the different angles, including the ANB angle (0.9°) in this example.

The skeletal class was determined from a CBCT using R2Gate™ software, version 2.0.0 (MegaGen, Daegu, Republic of Korea), by a single calibrated clinician (C.M.C.). Before analysis, a CBCT re-orientation option was used to align and center the patient’s CBCT and the Frankfort plane (drawn from Po—Porion to Or—Orbitale) was set as the horizontal plane. From the available options in the software (Figure 1a), an AP position of Mx Mn (antero-posterior position of the maxilla and mandible) analysis was selected. Several points were marked on the 2D sagittal middle section of the CBCT: nasion (N), sella (S), orbitale (Or), subspinale (A), upper incisor root apex (UIA), upper incisor incisal edge (UIT), lower incisor incisal edge (LIT), lower incisor root apex (LIA), supramentale (B) and pogonion (Pog) (Figure 1b). After setting the landmarks in the provided order, the software automatically calculated the A-Nasion-B (ANB) angle (Figure 2). The sagittal jaw relationship was classified using the ANB angle: normal skeletal class I (0.3° to +4.8°), skeletal class II (>+4.8°) and skeletal class III (<0.3°) [18,19,20].

### 2.2. Condylography

**Figure 3:**
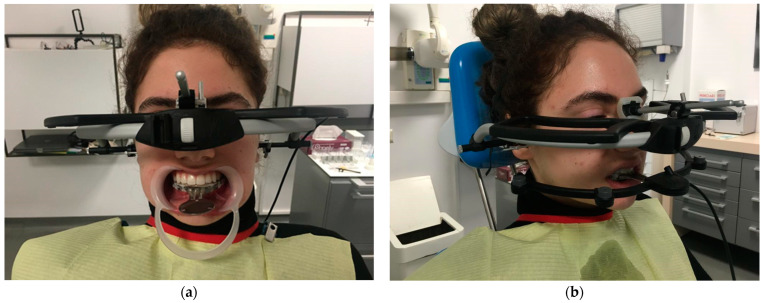
The set-up of ARCUSdigma™ 2 for condylography registration. Starting position: (**a**) front view and (**b**) lateral view.

Condylography was performed using the ultrasonic device ARCUSdigma™ 2 (Kavo, Biberach, Germany), based on a six-degrees-of-freedom concept [21] (Figure 3a,b). Before registration, a conventional impression of the maxillary and mandbular arch were taken using condensation-cured polymethyl siloxane impression material (Speedex, Coltene, Switzerland) of two consistencies (two phase): putty and light body, with Universal Activator and Coltene Adhesive in metal perforated stock trays (Zhermack SpA, Polesine, Italy). The impresions were poured with type IV Fujirock dental stone (GC Europe AG, Luzern, Switzerland) with added distilled water, according to the manufacturer’s instructions. The ARCUSDigma clutch was customized with Silatray (Siladent Dr. Böhme & Schöps GmbH, Goslar, Germany) photopolymerizable base plate to be applied paraocclusal, on the buccal side of the anterior lower dental arch [22].

On the day of registration, patients practiced the instructed mandibular movements (maximal opening, protrusive and lateral movements) before the actual condylography examination. The customized clutch previously checked intraorally (for the absence of contact with the upper teeth in an intercuspal position or in eccentric movements) was cemented on the lower arch with StructurPremium (Voco, GmbH, Cuxhaven, Germany) bi-acrylic composite.

During the condylography registration, each instructed movement began and ended from the starting position (reference position), with the mandible located in the maximally retrusive position in which lateral movements were possible, without dental contacts or applied force. All the movements of the patients during the examination were carefully observed by the operator and registered with the software KiD-Kavo (Kavo, Biberach, Germany). During the jaw kinematic recordings, patients had to make three protrusive, three left and three right lateral movements, and the software calculated the mean of the three recordings [17]. All the condylography registrations were performed by a single clinician (A.D.) according to the above-mentioned protocol recommended by the producer. The Camper plane (drawn from the inferior border of the ala of the nose to the superior border of the tragus [23]) was considered the reference plane [17,21,24].

### 2.3. Statistical Analysis

The collected data were entered into an Excel document and analyzed with IBM^®^ SPSS^®^ statistical software, v25.0 (IBM Corp., Armonk, NY, USA). A descriptive analysis was performed for the numerical values and mean, and the standard deviation (SD) was calculated. The left- and right-measured SCI values were assessed for a normal distribution (with Shapiro–Wilk test) and were further subjected to parametric tests. The significance was set at a *p*-value < 0.05. The paired *t* test was used to compare the mean difference pairwise (for both the right and left side); whereas the *t* test was used to compare the mean right and left SCI values among gender, age, dentate status, TMJ disfunction and parafunctional habits. A one-way ANOVA with a Bonferroni correction was used to determine statistically significant differences in the mean right and left SCI values depending on the skeletal class.

The sample size calculation was conducted based on previous studies [16,17] using G*Power (Version 3.1.9.7 software, Heinrich-Heine University, Dusseldorf, Germany). The calculate effect size (power 1-β) was 0.85 for a sample size of 130 individuals, for a probability (Type 1 error, α) of 0.05.

## 3. Results

One hundred and forty (one hundred females—71.4% and forty males—28.6%), aged between 19 and 66 years old, with a mean (SD) of 43.65 (±12.65), satisfied the inclusion criteria. For the entire analyzed group, the mean (SD) right SCI (SCI_R) was 34.68° (±12.44°) and the left SCI (SCI_L) was 34.94° (±13.23°), with an absolute difference [SCI-R–SCI_L] = 5.34° (SD ± 4.79°).

**Figure 4:**
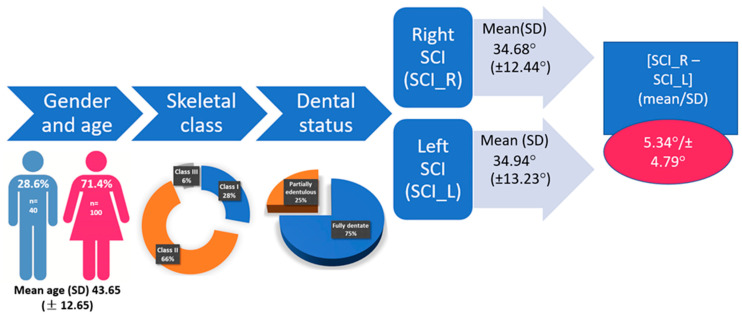
Summary of the characteristics of the enrolled participants. SCI_R = right sagittal condylar inclination and SCI_L = left sagittal condylar inclination.

The characteristics of the analyzed group are presented in Figure 4.

**Figure 5:**
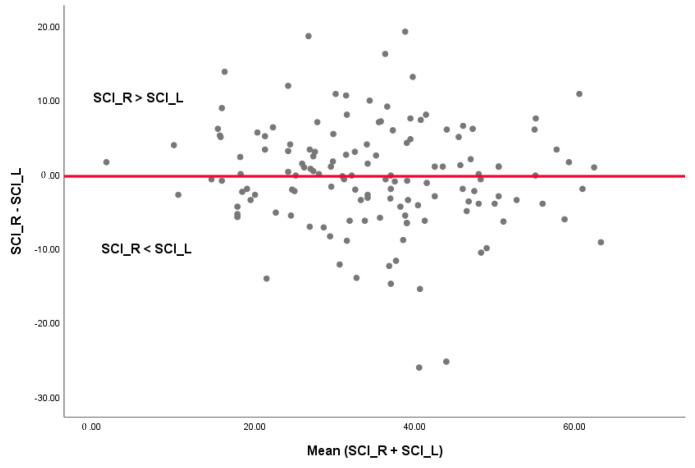
Differences between the left and right SCI values are plotted against the mean of SCI_R + SCI_L. SCI_R = right sagittal condylar inclination and SCI_L = left sagittal condylar inclination. The points represent the difference for each enrolled patient.

**Figure 6:**
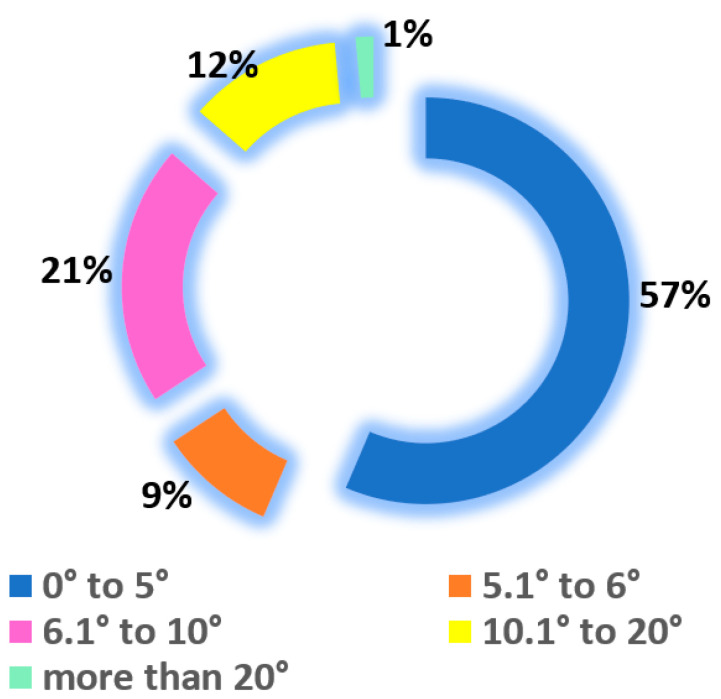
Absolute differences between the left and right SCI are grouped into five categories, according to the absolute value of [SCI_R–SCI_L]. SCI_R = right sagittal condylar inclination and SCI_L = left sagittal condylar inclination.

No statistically significant differences were observed between the right and left SCI (*p* = 0.67). In Figure 5, the differences between SCI_R and SCI_L are plotted against the mean value of SCI_R + SCI_L. The 95% CI is [−14.33,13.80] with values between 0° and 26.1° and a mean absolute value of 5.34° (Figure 4). The absolute values of [SCI_R–SCI_L] grouped in intervals are displayed in Figure 6. It can be noted that 57% of the values are between 0° and 5°.

The right- and left-mean SCI values and differences for gender and skeletal class are displayed in Table 1 and Table 2, respectively.

A statistically significant difference in the left SCI value was observed when comparing the skeletal classes (Table 2). When a post hoc Bonferroni correction was applied to a one-way ANOVA, the skeletal class III had statistically significant differences in the SCI_L values when compared to those of skeletal classes I and II (*p* < 0.05). No significant differences were observed between skeletal classes I and II.

The right- and left-mean SCI values analyzed when taking dental status into consideration are presented in Table 3. A statistically significant higher value was obtained for the right SCI in the partially edentulous group. However, higher values were registered for the same group for the left SCI, but were not statistically significant (Table 3).

The right- and left-mean SCI values analyzed when taking into consideration the occurrence of TMJ disorders and parafunctional habits are presented in Table 4 and Table 5, respectively. No statistically significant differences were noticed when these two factors were considered. However, a slightly higher value was registered for both the left and right SCI for patients with TMJ disorder (Table 4).

## 4. Discussion

The registration of individual articular parameters, such as SCI, Bennett angle and immediate side shift (ISS), is the first step for analyzing and planning any oral rehabilitation, and for maintaining and restoring essential functions, such as mastication, speech, deglutition and esthetics.

Among the above-mentioned parameters, the SCI, defined as the angle formed between the protrusive condylar path and the Frankfort plane [25,26], or another horizontal reference plane such as Camper’s plane [14,23] or the axis-orbital plane [27], mostly influences dynamic tooth morphology [14,16]. Therefore, the determination of the individual value of the SCI will ensure better diagnosis, treatment planning and more accurate prosthetic restorations, saving valuable clinical time relating to adjustments of occlusal interferences.

Several clinical, radiological and instrumental methods have been proposed for condylar path registration, but few studies have analyzed the intraindividual values of the left and right SCI based on skeletal class [17]. Semi-adjustable articulators, mostly used in clinical settings, are usually programmed with average arbitrary values, or by using symmetrical individual static records, most of the time not simulating the patient’s TMJ anatomy. Most of the virtual articulators, part of the computer-aided design (CAD) software, use mathematical algorithms which basically reproduce the mechanical articulators used for customizing occlusal anatomy [26], but do not mimic the actual condylar movements.

In our study, we focused on the SCI registered for the right and left TMJ for different skeletal classes, based on the ANB angle, to identify if the values were similar and if statistically significant differences could be noticed depending on gender, skeletal class, dental status, TMJ disorders or parafunctional habits. The reference plane considered by the measuring device was Camper’s plane, differing from the Frankfort horizontal, between 9° and 15° [17,28]. The mean values obtained for the right and left SCI were 34.68° and 34.94°, respectively. These values are lower than those obtained by Cimic et al. in their study of 51 subjects with an Angle’s class I occlusion [17]. The authors used the same reference plane (Camper) and same measuring device (ARCUSdigma™ 2), but they divided the left and the right condyle path in the sagittal direction into three equal sequences, based on the whole condylar path length, and the software calculated the SCI as the angle between Camper’s plane and each sequence of the condylar path. For sequence 1, considered by the authors to be used for articulator setup, the values were 46.8° for the right SCI and 45.9° for the left SCI; for sequence 2 the values were 39.3° (right SCI) and 39.4° (left SCI); for sequence 3 the values were 22.9° (right SCI) and 22.4° (left SCI) [17]. They reported the mean differences between the left and right SCI as 5.7° for sequence nos. 1 and 2 and 6.0° for sequence no. 3. These mean values are similar to those obtained in our study (Figure 4) of 5.34° (±4.79) for all the enrolled patients. However, the mean value for skeletal class I in our group was slightly lower at 4.91 (±4.18), maybe due to the fact that those authors used Angle’s dental classification and we used the ANB angle to define the skeletal classes.

Das et al., in a study of 40 healthy participants (20 females and 20 males) using a clinical (protrusive interocclusal record) and a radiographical method (CBCT), obtained mean values of 32.78°/32.90° (SCI_R/SCI_R) and 35.43°/35.18° (SCI_R/SCI_R), respectively, but used Frankfort as the reference plane. The mean values obtained based on the CBCT measurements were slightly higher than those obtained using the clinical method, but without statistically significant differences [16]. The authors did not find any statistically significant differences between the left and right SCI and they did not define the skeletal class of the study participants [16].

The average SCI, according to the literature, is between 20° and 33° [29]. The differences in SCI values are dependent on the reference plane, the method used, the measuring instruments and also the characteristics of the analyzed group. However, the recommended average settings differ between manufactures of the semi-adjustable articulators; for example, a SCI of 30° is the recommendation for the virtual and analog Artex^®^CR (Amann Girrbach AG, Koblach, Austria), with Camper as the reference plan [14,30], and the same settings are recommended for Stratos° 100 (IvoclarVivadent, Schaan, Lichtenstein) [21].

In the present study, we did not notice any predominance of greater values of the SCI for the right or left TMJs, as shown in Figure 5. The difference was calculated as the absolute value: [SCI_R–SCI_L] = [(SCI_R–SCI_L) if SCI_R > SCI_L] or [(SCI_L–SCI_R) if SCI_R < SCI_L]. More than half of the participants (57%) had a difference of 5° or less between the right and left SCI, which was reported as normal [31], but 13% had a difference between the right and left TMJ of more than 10° (Figure 6), the maximum registered value being 26.1° for a skeletal class II patient. For Angle class I subjects, Cimic et al. reported a maximal variation between the left and right side of 25° [17].

The differences between the left and right SCI values in our study were not statistically significant, agreeing with other studies [16,32]. However, studies by Zamacona et al. [33] and by El Gheriani and Winstanley [34] reported significant differences between the left and right SCI.

No statistically significant differences were noticed between the left and right SCI when the patient’s gender was considered. This could be due to the greater number of female (n = 100) compared to male patients (n = 60) enrolled and is one of the limitations of the present study. Overall, despite of the lack of statistical significance, the mean left and mean right SCI were greater in males than females: SCI_L 36.89 (±12.67) vs. 34.16 (±13.43) and SCI_R 37.68 (±12.23) vs. 33.48 (±12.37) (Table 1), in accordance with other studies [16].

When the right and left SCI were analyzed based on skeletal class, a statistically significant difference was noticed for the left SCI between class III vs. class I and class III vs. class II. Lower mean values of the SCI were also registered for the right SCI, without being statistically significant. In fact, the class III subjects registered the lowest mean SCI compared to those of class I and class II: SCI_R 27.84 (±11.74) vs. 35.10 (±10.03) for class I and 34.79 (±13.29) for class II, and SCI_L 22.69 (±8.58) vs. 35.87 (±11.10) for class I and 35.75 (±13.92) for class II (Table 2). The lowest mean SCI values for class III, and statistically significant differences for both the left and right SCI between class III vs. class I, and class III vs. class II, was reported by Lewandowska et al. in a similar study including 75 patients (52 females and 23 males), but using the electronic condylograph Cadiax Compact for mandibular path recording [35]. The lowest mean SCI for skeletal class III compared to classes I and II, similar to our study, was also reported by Zimmer et al. [36] in a study of 57 non- orthodontically treated patients and by Canning et al. in a study of 73 young subjects [37]. However, Canning and co-authors reported for the class II subjects statistically significant higher SCI values compared to those of class I (*p* < 0.05) and class III (*p* < 0.001) [37], different from our results. However, in all the above-mentioned studies there was a lack of standardization in determining the skeletal class. For instance, Lewandowska et al. [35] used the cephalometric analysis without a clear description of the methodology, Canning et al. [37] used the patient’s profile photographs examined by three orthodontists and three prosthodontists and Zimmer et al. [36] used the dental relationship to define the patient’s class.

The dentate status also influenced the mean left and right SCI and a statistically significant difference was observed for the mean right SCI (Table 3). Lower mean values were registered for the left and right SCI for the fully dentate patients compared to the partially edentulous patients. This is not unusual and is due to the enrolment protocol. All the fully dentate participants were seeking orthodontic treatment and the partially edentulous participants were seeking dental restorations. The CBCT used for skeletal class diagnosis were performed only for future treatment purposes (orthodontic or dental implants insertion), so the fully dentate participants were mainly classes II and III. This was one of the limitations of the present study.

TMJ disorder and parafunctional habits did not influence SCI values to a statistically significant degree.

The present study has some limitations. First, the groups were not homogenous; there were more female patients (n = 100) are than male patients (n = 40). This was mainly due to the higher percentage of female patients who seek dental services, especially for esthetic reasons, in our country. In addition, there were more class II (n = 92) participants than class I (n = 39) and class III (n = 9). This last fact could explain the lower SCI mean value compared to a similar study which only included class I participants [21]. The enrollment of patients in the present study was based on treatment requirements. This explains the greater number of fully dentate patients (75%) as well as the percentage of class II and class III skeletal anomalies (overall 72% of the subjects). Future prospective clinical trials with a similar protocol and a homogenous group of participants are required to verify the findings of the present study.

For the patients requiring orthodontic treatment, condylography is recommended as part of the diagnostic protocol due to the requirement to evaluate the mandibular kinematic and asymmetry in the left vs. right TMJ, as can be noted from the findings of the present study.

The goal of our study was to evaluate the left and right SCIs and to see if the consideration of a mean equal value for both TMJs was accurate for diagnosis, treatment planning and the manufacturing of fixed or removable restorations. The results of our study demonstrated a great interindividual and intraindividual variety in SCI values and the requirement to individually register the TMJ parameters for a personalized treatment.

## 5. Conclusions

For an optimal functionalization of prosthetic restorations and for an ideal treatment plan, the registration of both the left and right path of the condyles and the articular disc should be taken into consideration.

Differences of up to 26.1° between the right and left SCI were not uncommon, and intraindividual differences were noticed, although they were not statistically significant.

For the analyzed group, the mean values of the right and left SCI for all the skeletal classes were 34.68° (±12.44°) and 34.94° (±13.23°), respectively.

The lowest mean SCI value was obtained for the skeletal class III participants, and was statistically significant for the left SCI.

## Figures and Tables

**Table 1:** Comparison of right and left SCI by gender.

	Gender	Mean (SD)	Mean Difference	95% Confidence Interval of the Difference	t Value (df)	*p* Value
SCI right (SCI_R)	Male (n = 40)	37.68 (±12.23)	4.20	−0.37 to 8.76	1.82 (138)	0.07
	Female (n = 100)	33.48 (±12.37)				
SCI left (SCI_L)	Male (n = 40)	36.89 (±12.67)	2.73	−2.16 to 7.62	1.10 (138)	0.27
	Female (n = 100)	34.16 (±13.43)				
[SCI_R–SCI_L]	Male (n = 40)	4.92 (±4.44)	−0.59	−2.37 to 1.18	0.66 (138)	0.51
	Female (n = 100)	5.50 (±4.93)				

SCI_R = right sagittal condylar inclination; SCI_L = left sagittal condylar inclination; [SCI_R–SCI_L] = (SCI_R–SCI_L) if SCI_R > SCI_L or (SCI_L–SCI_R) if SCI_R < SCI_L; df = degree of freedom; *p* < 0.05 shows statistical significance.

**Table 2:** Comparison of right and left SCI by skeletal class.

	Skeletal Class	Mean (SD)	*p* Value
SCI right (SCI_R)	Class I (n = 39)	35.10 (±10.03)	0.207
	Class II (n = 92)	34.79 (±13.29)	
	Class III (n = 9)	27.84 (±11.74)	
SCI left (SCI_L)	Class I (n = 39)	35.87 (±11.10)	0.02 *
	Class II (n = 92)	35.75 (±13.92)	
	Class III (n = 9)	22.69 (±8.58)	
[SCI_R–SCI_L]	Class I (n = 39)	4.91 (±4.18)	0.75
	Class II (n = 92)	5.44 (±4.95)	
	Class III (n = 9)	6.13 (±5.90)	

SCI_R = right sagittal condylar inclination; SCI_L = left sagittal condylar inclination; [SCI_R–SCI_L] = (SCI_R–SCI_L) if SCI_R > SCI_L or (SCI_L–SCI_R) if SCI_R < SCI_L; * statistically significant.

**Table 3:** Comparison of right and left SCI by dental status.

	Dental Status	Mean (SD)	Mean Difference	95% Confidence Interval of the Difference	t Value (df)	*p* Value
SCI Right (SCI_R)	Fully dentate (n = 105)	33.19 (±12.43)	−5.96	−10.67 to −1.25	−2.50 (138)	0.01 *
	Partially edentulous (n = 35)	39.15 (±11.51)				
SCI left (SCI_L)	Fully dentate (n = 105)	33.74 (±13.56)	−4.80	−9.86 to −0.26	−1.88 (138)	0.06
	Partially edentulous (n = 35)	38.54 (±11.65)				
[SCI_R–SCI_L]	Fully dentate (n = 105)	5.70 (±4.88)	1.44	−0.40 to 3.27	1.55 (138)	0.13
	Partially edentulous (n = 35)	4.26 (±4.39)				

SCI_R = right sagittal condylar inclination; SCI_L = left sagittal condylar inclination; [SCI_R–SCI_L] = (SCI_R–SCI_L) if SCI_R > SCI_L or (SCI_L–SCI_R) if SCI_R < SCI_L; df = degree of freedom; * statistically significant.

**Table 4:** Comparison of the right and left SCI by occurrence of temporomandibular disorder.

	TMJ Disorder (Yes/No)	Mean (SD)	Mean Difference	95% Confidence Interval of the Difference	t Value (df)	*p* Value
SCI Right (SCI_R)	No (n = 92)	33.40 (±12.49)	−3.72	−8.07 to 0.63	−1.69 (138)	0.09
	Yes (n = 48)	37.12 (±12.09)				
SCI left (SCI_L)	No (n = 92)	33.80 (±13.91)	−3.32	−7.96 to 1.33	−1.41 (138)	0.16
	Yes (n = 48)	37.12 (±11.65)				
[SCI_R–SCI_L]	No (n = 92)	5.39 (±5.15)	0.15	−1.54 to1.84	0.17 (138)	0.86
	Yes (n = 48)	5.24 (±4.05)				

SCI_R = right sagittal condylar inclination; SCI_L = left sagittal condylar inclination; [SCI_R–SCI_L] = (SCI_R–SCI_L) if SCI_R > SCI_L or (SCI_L–SCI_R) if SCI_R < SCI_L; df = degree of freedom; *p* < 0.05 shows statistical significance.

**Table 5:** Comparison of right and left SCI by occurrence of parafunctional habits.

	Parafunctional Habits (Yes/No)	Mean (SD)	Mean Difference	95% Confidence Interval of the Difference	t Value (df)	*p* Value
SCI Right (SCI_R)	No (n = 106)	34.36 (±12.14)	−1.33	−8.18 to 3.53	−0.54 (138)	0.59
	Yes (n = 34)	35.68 (±13.44)				
SCI left (SCI_L)	No (n = 106)	34.85 (±13.24)	−0.37	−5.54 to 4.80	−0.14 (138)	0.89
	Yes (n = 34)	35.22 (±13.39)				
[SCI_R–SCI_L]	No (n = 106)	5.50 (±5.12)	−0.66	−1.21 to 2.53	0.70 (138)	0.49
	Yes (n = 34)	4.84 (±3.57)				

SCI_R = right sagittal condylar inclination; SCI_L = left sagittal condylar inclination; [SCI_R–SCI_L] = (SCI_R–SCI_L) if SCI_R > SCI_L or (SCI_L–SCI_R) if SCI_R < SCI_L; df = degree of freedom; *p* < 0.05 shows statistical significance.

## Data Availability

Data is available by request from the corresponding author.
